# Supplementary material for: Online sexual health survey implementation: Practical lessons from a comparative, mixed-methods analysis of 30 countries
Source: PLoS One. 2026 Jun 3;21(6):e0346722. doi: 10.1371/journal.pone.0346722 (PMC13232842; doi:10.1371/journal.pone.0346722)
Supplement: Attachment — Submitted filename: Response to Reviewers.docx [file pone.0346722.s002.docx]

Dear Ivan Alejandro Pulido Tarquino,

Thank you for providing helpful comments on our manuscript. We have used them to strengthen the manuscript.

Find below point-by-point responses.

Sincerely,

Hanna and Joe on behalf of the author team

| Review comment (reviewer) | **Comment** | **Action taken** |
| --- | --- | --- |
| 1. Please ensure that your manuscript meets PLOS ONE's style requirements, including those for file naming. | We agree that there were some stylistic issues not in line with journal requirements. | 1. Figure headings changed to align with the guide. (line 162 - 163 and 364 - 366)  2. References changed to align with the guide (see lines 791 -919).  3. Table reformatted to align with the guide. See lines 124, 198 and 209) |
| Your ethics statement should only appear in the Methods section of your manuscript. If your ethics statement is written in any section besides the Methods, please delete it from any other section. (Editor) | We agree that the ethics statement should be part of the methods section. | We subsumed ethics into the methods section, giving it a subheading (level 2) instead of its own heading (level 1). See lines 169 – 176. |
| Instead of the last sentence in the abstract, you should give some hints from your recommendations (lines 54-57). (R1)  Abstract: Revise it as per review suggestion, you would resume the implications of your study findings.  (editor) | We agree that adding recommendations in the abstract would be useful. | **Deleted:** The identified themes are relevant for those organising online sexual health research.  **Replaced with:** Our study suggests the need for greater consideration of bias related to communication, especially the digital divide, when designing and implementing online surveys. (see lines 54-56) |
| Content analysis should be described with more details: how many persons performed these (in parallel?) and how did you handle differing interpretations? (R1)  Methods: Provide more detail on the process, including how many persons performed the analysis (simultaneously or independently), and how differing interpretations were resolved, highlighting what measures do you implement to maintain trustworthiness of your study findings. (Editor) | We agree that more details in this section would be useful. | Elaboration provided lines 149 and 156 – 161 (inclusive). |
| You should also mention the number of persons eligible per country since they are quite different (for example: China vs Luxembourg).  Study population: Clarify the number of persons eligible per country, noting the large differences as mentioned by the reviewer. | While we agree that this information would be helpful, not all studies had a list of the entire population of eligible participants. As a result, we have added this to the list of limitations. | See lines 768 – 773 in the strengths and limitations section. |
| Discussion is very lengthy and should not replicate too many details from results. Instead, you should draw comparisons to other studies with the theme of sexual health. Can you- in this context- come back to the fact that in most countries much more females than males answered to the survey? (R1)  This section would provide comparisons of your results with other studies, especially those on sexual health, and reflect on the gender imbalance in responses (notably the higher participation of females).  (Editor) | We agree that the discussion was too long. We have narrowed and focused the discussion. | See track changes throughout the discussion section (lines 367 – 762, noting that there is an error in continuity where the line after 474 jumps to 723).  Specific reference to the gender differences is now added into lines 450 to 452. |
| I cannot read Figure 1- reproduction is bad (R1)  Figures and tables: Improve the overall quality of figures and tables to ensure readability. (Editor) | Apologies for the poor resolution – we have revised Figures 1 and 2. | The authors have revised and improved the image quality of Figures 1 and 2. Tables were checked for quality, and these were acceptable as is. |
| line 84: you should write it's or the instead of it | Sorry for the typo. | Changed it to its (line 92 in revised/track changes draft) |
| line 94/95: What do you want to say with: The survey was designed to capture a broad cross-section of the adult population 15. which age range is meant? | We agree that the age inclusion criteria are important. | Added: The eligibility criteria were that respondents must be aged ≥18 years (although some countries included ethical provisions to enable younger people to participate), reside in a participating country, be able to read the survey language, and have access to the survey online, providing informed consent [28]. (see lines 103 – 106) |
