# Supplementary material for: Online sexual health survey implementation: Practical lessons from a comparative, mixed-methods analysis of 30 countries
Source: PLoS One. 2026 Jun 3;21(6):e0346722. doi: 10.1371/journal.pone.0346722 (PMC13232842; doi:10.1371/journal.pone.0346722)
Supplement: Attachment — Submitted filename: Response to Reviewers 13 April 2026.docx [file pone.0346722.s004.docx]

Dear Dr. Pruller,

Thank you for providing helpful comments on our manuscript. We have used them to strengthen the manuscript.

Find below point-by-point responses. Please note line numbers correspond to the Tracked Changed document in red and the clean document in black.

Sincerely,

Hanna and Joe on behalf of the co-authors

| Review comment (reviewer) | **Comment** | **Action taken** |
| --- | --- | --- |
| Reviewer #1: | | |
| Many thanks- I can now read now fig 2 after downloading it separately, and I have no further remarks. Interesting article | The authors thank Reviewer 1 for their time in reviewing our paper and for their insightful and helpful comments. | NA |
| Reviewer #2: | | |
| 1. The authors might reflect on the role of online surveys more specifically to sexual health. Did any countries report that people were more likely to take part in an online survey about sexual health because it could be viewed as more private? Overall, the paper focuses a lot on the online methodology, but less about how this interacts with the survey content, which can be quite sensitive. If these sorts of discussions did not come up, the authors might list this as a limitation or area of future research. | We agree that this could be made more explicit. We have added some information to highlight this in Theme 3 and in the discussion. | See lines 343-347 in the tracked version (319- 323 in the clean version) and 423-430 (391 – 398) |
| 2. The authors may wish to reference, in the introduction or discussion, the Natsal-COVID study in the UK, which achieved a quasi-representative study based on comparisons to census data. Results and methods publications for this study are readily available. The authors discuss issues with representativeness in the included studies, but do not explain how representativeness was measured. This would be helpful to add. <https://wellcomeopenresearch.org/articles/7-166> | We agree that mentioning Natsal-COVID would be useful and flagged this study in the discussion. At the same time, a broader discussion about metrics for representativeness and how best to measure this is beyond the scope of this manuscript. | Lines 379-380 (367-368) |
| 3. In lines 145-146, there seems to be an issue with the referencing software. | The authors could not identify any issues with the references in these lines. | No action taken – please refer to screenshots below this table* |
| 4. The authors may wish to more explicitly explain how their findings can inform future sexual health research post-pandemic, particularly in the context of survey fatigue. | We agree that this could be made more explicit and have added some sentences to reflect this. | See lines 446 – 453 (416 - 423) |

*Screenshot taken from Manuscript with Track Changes

Screenshot taken from Manuscript with No Track Changes
